# Deciphering Optimal Radar Ensemble for Advancing Sleep Posture Prediction through Multiview Convolutional Neural Network (MVCNN) Approach Using Spatial Radio Echo Map (SREM)

**DOI:** 10.3390/s24155016

**Published:** 2024-08-02

**Authors:** Derek Ka-Hei Lai, Andy Yiu-Chau Tam, Bryan Pak-Hei So, Andy Chi-Ho Chan, Li-Wen Zha, Duo Wai-Chi Wong, James Chung-Wai Cheung

**Affiliations:** 1 Department of Biomedical Engineering, Faculty of Engineering, The Hong Kong Polytechnic University, Hong Kong 999077, China; 2 Research Institute of Smart Ageing, The Hong Kong Polytechnic University, Hong Kong 999077, China

**Keywords:** radar, sleep posture, sleep apnea, sleep medicine, polysomnography, ubiquitous health

## Abstract

Assessing sleep posture, a critical component in sleep tests, is crucial for understanding an individual’s sleep quality and identifying potential sleep disorders. However, monitoring sleep posture has traditionally posed significant challenges due to factors such as low light conditions and obstructions like blankets. The use of radar technolsogy could be a potential solution. The objective of this study is to identify the optimal quantity and placement of radar sensors to achieve accurate sleep posture estimation. We invited 70 participants to assume nine different sleep postures under blankets of varying thicknesses. This was conducted in a setting equipped with a baseline of eight radars—three positioned at the headboard and five along the side. We proposed a novel technique for generating radar maps, Spatial Radio Echo Map (SREM), designed specifically for data fusion across multiple radars. Sleep posture estimation was conducted using a Multiview Convolutional Neural Network (MVCNN), which serves as the overarching framework for the comparative evaluation of various deep feature extractors, including ResNet-50, EfficientNet-50, DenseNet-121, PHResNet-50, Attention-50, and Swin Transformer. Among these, DenseNet-121 achieved the highest accuracy, scoring 0.534 and 0.804 for nine-class coarse- and four-class fine-grained classification, respectively. This led to further analysis on the optimal ensemble of radars. For the radars positioned at the head, a single left-located radar proved both essential and sufficient, achieving an accuracy of 0.809. When only one central head radar was used, omitting the central side radar and retaining only the three upper-body radars resulted in accuracies of 0.779 and 0.753, respectively. This study established the foundation for determining the optimal sensor configuration in this application, while also exploring the trade-offs between accuracy and the use of fewer sensors.

## 1. Introduction

Sleep posture is one of the essential components in sleep tests and sleep monitoring systems that provide valuable insights into sleep patterns and sleep-related health [1,2]. Various health conditions and their treatments have been found to correlate with sleep positions or sleep postures [3,4]. Sleep posture is related to the biomechanics of the airway and spine, implicating sleep-related breathing and musculoskeletal disorders [5]. For instance, adopting a lateral sleep posture can alleviate the symptoms of sleep apnea [3], while a supine posture may provide relief for individuals suffering from lower back and neck pain [4]. Sleep posture also serves as an indicator for sleep quality and sleep ergonomics [6,7]. It has been observed that individuals with a poor sleep quality frequently change postures and prefer the supine position [6]. In other words, sleep posture serves as a critical link in the complex relationship between sleep health, quality, and ergonomics [8]. Comprehensive overnight sleep studies require objective and efficient sleep posture measurements to inform personalized sleep recommendations and interventions. However, traditional sleep studies (i.e., polysomnography) rely on manual observation to identify sleep postures, which is labor-intensive and may be prone to errors [2]. To address this challenge, specialized sensors and artificial intelligence for sleep posture measurement and estimation have become increasingly prevalent. These technologies enable the automatic and accurate acquisition of sleep posture, thus enhancing the precision and efficiency of sleep studies.

Contact and non-contact sensors are two major categories in sleep posture recognition technologies. Numerous studies have applied pressure sensors and wearable devices to estimate sleep posture. Pressure mapping technologies, which integrate sensitive conductive sheets into mattresses or bedsheets, can identify different sleep postures based on changes in the body’s interfacial pressure patterns [9,10]. However, they might be costly, have limited availability, be influenced by specific mattresses [11,12], and require regular maintenance and cleaning. Furthermore, wearable sleep technologies, also known as actigraphy, incorporate various sensors to measure both biophysical signals and sleep postures [13,14,15], in addition to sleep stage classification. They can also be readily usable at home [16,17]. Accelerometers within these wearable devices can be attached to the body to identify sleep postures and track posture changes [18,19,20]. Nevertheless, despite the lack of clear evidence on this issue [21], it is believed that some users, especially older people or those with emotion problems, may find actigraphy uncomfortable to wear and difficult to comply with [22]. On the other hand, machine learning and deep learning models, particularly support vector machines (SVMs) and convolutional neural networks (CNNs), have been applied to facilitate sleep posture estimation with these devices [2,23].

Non-contact methods utilize optical sensors, particularly video cameras and computer vision systems, to estimate sleep posture [14,15,24]. It is becoming increasingly common to independently utilize depth or infrared cameras or use them to complement traditional video cameras for sleep posture estimation. Their strength lies in the ability to function in night-time conditions and protect privacy [25,26,27,28,29,30], and they are also used to monitor bed-exiting events [31,32]. Another significant advantage of depth cameras is their ability to estimate sleep posture under-blanket by assessing the depths of the images, which optical cameras cannot perform [27,28,29,33]. These techniques are often combined with machine or deep learning models, such as CNNs and SVMs [27,28,29], to automate the process. In particular, Tam et al. [28] proposed an intraclass mix-up technique to generalize blanket conditions, and efforts have been made to estimate the joint coordinates in sleep postures [28,34].

Radar technology presents another alternative non-contact method for sleep posture estimation. It combines the advantages of depth cameras and requires even less exposure and visuals for accurate estimation [35]. Several studies have explored the potential of radar technology for sleep posture recognition along with machine or deep learning models. Higashi, et al. [36] achieved an accuracy of 88% using 24 GHz Doppler radar data and machine learning for sleep posture recognition. BodyCompass, a system developed by Yue, et al. [37], integrates FMCW radar with a sweeping frequency ranging from 5.4 GHz to 7.2 GHz to determine patient posture, achieving an accuracy of up to 94%. Kiriazi, et al. [38] investigated sleep posture estimation using a dual-frequency Doppler radar emitting 2.4 GHz and 5.8 GHz waves mounted on the ceiling above the bed to monitor torso reflections and movement. However, the use of continuous wave radar for indoor sleep monitoring might be limited due to potential multipath interference [39]. To address this challenge, Piriyajitakonkij, et al. [40] proposed a method that utilizes data from both temporal and spectral domains, enhancing IR-UWB signal detection for the recognition of four sleep transitional postures.

Sleep posture recognition can be considered a fine-grained classification problem, which can be effectively addressed using multimodal data fusion or multiview data fusion approaches. These methods integrate diverse data sources or multiple views of the same data to enhance the discriminative power of classification models. For example, Khaire, Imran, and Kumar [41] demonstrated that fusing RGB-D and skeletal data to improve human activities classification by providing complementary information enhanced feature representation. Similarly, Zhu and Liu [42] employed multiview attention to combine visual and optical flow data for fine-grained action recognition, achieving a superior accuracy compared to single-modality approaches. XIE et al. [43] utilized co-located IR-UWB radar and depth sensors for fine-grained activity recognition and tracking in a domestic setting. These advancements highlight the potential of data fusion approaches to tackle the challenges associated with fine-grained classification by leveraging the strengths of multiple data modalities.

The number and placement of sensors are important factors in the performance of sleep posture estimation. While intuitively increasing the number of sensors and diversifying their positions could enhance performance, this would introduce additional costs and complexities into the experimental setup. Moreover, it could potentially burden the model for posture estimation, since it would need more computing resources to process more sensor data.

The research gap lies in the insufficient understanding of the minimal sensor configuration and placement strategy required to achieve accurate results. Existing studies often presume placing the radar in the center as a rule of thumb and focus on optimizing predictions based on this configuration. Our research question involves determining the minimum number of radar sensors and their positions to achieve the best performance in sleep posture estimation. Our previous studies have explored multiple radar configurations, including dual (examining single-radar settings and dual-radar settings) and triple settings (examining the influence of top, head, and side radar placement combinations) [44,45]. In this study, we aim to evaluate the performance of sleep posture estimation using different arrangements and combinations of eight radar sensors. Another innovation of this study is the proposal of a data fusion technique for the multiple radar configuration. This technique is designed to facilitate more efficient processing by deep learning models and to improve the effectiveness of sleep posture estimation. We first applied deep learning models, including ResNet, EfficientNet, DenseNet, PHResNet, Residual attention network (attention-56), and Swin Transformer, to the data of all radar sensors. Subsequently, we experimented with different arrangements and combinations of the radar sensors for the model that demonstrated the best performance. The main contributions of this study include, as follows:

- Incorporating a Multiview Convolutional Neural Network (MVCNN) architecture to leverage deep feature extractors for precise sleep posture estimation.
- Introducing Spatial Radar Echo Maps (SREMs) to enhance radar-based sleep posture prediction.
- Identifying the optimal radar sensor configuration for an improved posture estimation accuracy.

## 2. Materials and Methods

### 2.1. System Setup

This study employed the Impulse-radio ultra-wideband (IR-UWB) radar. IR-UWB radars transmit short-duration impulse signals using a transmitter. When the emitted radar pulse encounters an object, the transmitted pulse partially penetrates the object, while the remainder is reflected back by the receiver. The Time of Arrival (TOA) of the reflected pulse is measured to determine the distance between the target and the radar. Mathematically, the received signal can be expressed as the formula in Equation (1).

(1) $$y\left(t\right)=\sum_{i=1}^{P}A_{i}\delta \left(t-\tau_{i}\right)+n(t)$$

where *P* is the number of multipaths, $A_{i}$ is the amplitudes associated with the multipaths, $\tau_{i}$ is the time delay of the multipath components, and n(t) represents the noise captured from random variations and disturbances in the channel.

We utilized eight IR-UWB radar sensors, which are integrated system-on-chips (Xethru X4M03 v5 from Novelda, Oslo, Norway) operating at a center frequency of 7.29 GHz and a bandwidth of 1.4 GHz. They comprise two key components: a programmable controller and an antenna. On the receiving end, the system boasts a high sampling rate of 23.328 GS/s and a total radar frame length of 9.87 m. This represents a distance resolution of 0.00643 m between each data point received by the radar. Additionally, the receiver maintains a sufficient gain of 14.1 dB and a low noise figure of 6.7 dB. Both the elevation and azimuth angles of the radars spanned a wide range from −65° to +65°. Table 1 shows the parameters used in this study. Notably, the detection range of the radars was set to encompass the area of interest (RoI) of our study.

### 2.2. Radar Placement

**Figure 1:**
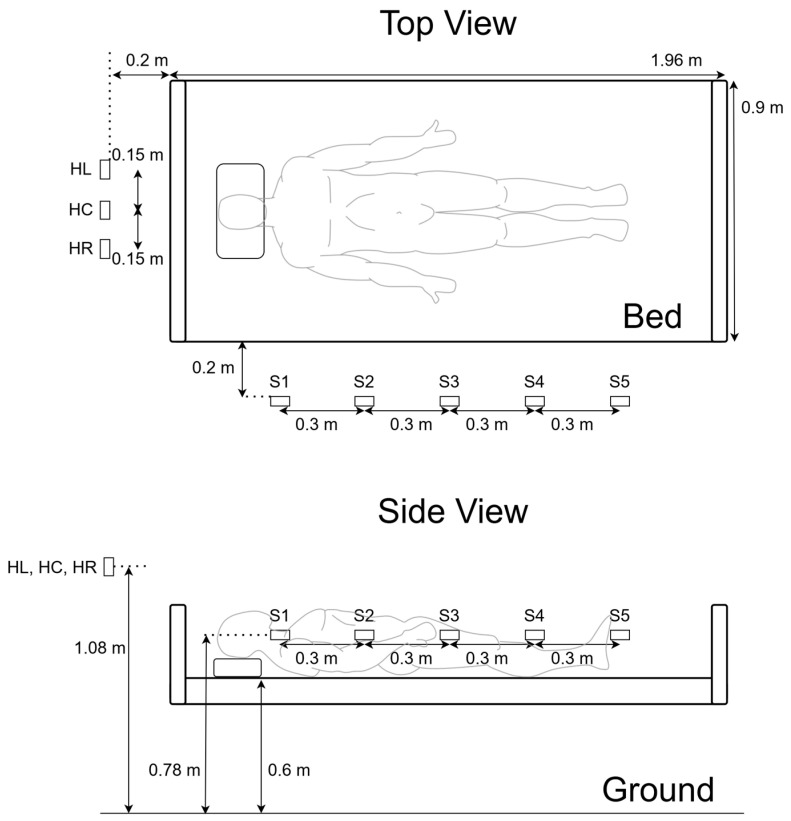
Radar placement around the bed. S1–S5 denote radar sensors arranged from cranial (S1) to caudal direction. HL, HC, and HR denote radar positions at the left, center, and right of the headboard.

The radars were positioned around a bed of 196 cm × 90 cm × 60 cm (length, width, and height). Five radars were positioned on the side of the bed, 78 cm from the ground, shooting on the body, torso, and limbs at 15 cm interval spacings. They are abbreviated as *S*_1_, *S*_2_, *S*_3_, *S*_4_, and *S*_5_, from cranial to caudal. Three radars were placed at the headboard side of the bed, 108 cm from the ground, spanning over the shoulders and the head, which were positioned higher than the side radars to avoid blockage of the headboard. They are abbreviated as *H_L_*, *H_C_*, and *H_R_*, corresponding to left, central, and right, respectively. To ensure compatibility with scenarios where participants might extend their limbs beyond the edges of the bed and accidentally hit the radars, all radars were positioned 20 cm from the edges with the aforementioned height parameters for practicality. We first assessed the impact of the head radars. Subsequently, we decided to maintain the use of the central radar due to its extensive coverage and alignment with our existing study. Following this, we evaluated the influence of the side radars. The configuration of the sensor placements, along with their labels, is illustrated in Figure 1.

### 2.3. Experiment Protocol and Data Collection

A total of 70 adults (39 males and 31 females) were recruited from a university to participate in this experiment. The inclusion criteria were adults aged over 18. The exclusion criteria were people with the absence of any limbs or pregnancy. People who had difficulty staying in or positioned in a specific position in bed were also excluded. The study was approved by the Institutional Review Board of The Hong Kong Polytechnic University (Reference Number: HSEARS20210127007). Before the experiment began, all participants received a thorough explanation of the procedures, both orally and in writing. Informed consent was obtained from all subjects involved in the study.

The average age of the enrolled participants was 26.3 years (standard deviation: 11.3 years, ranging from 18 to 67). Their average height was 168.2 cm (standard deviation: 8.32 cm, ranging from 150 cm to 186 cm) and their average weight was 64.0 kg (standard deviation: 12.3 kg, ranging from 43 kg to 108 kg). The average BMI was 22.6 (standard deviation: 4.03, ranging from 16.3 to 43.8)

**Figure 2:**
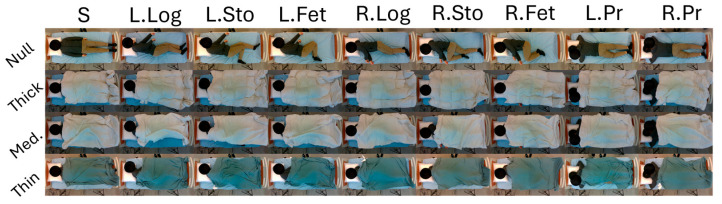
Illustration of the nine sleep postures with three blanket conditions (thick, medium, and thin). The postures are: supine (S); left lateral side lying with both legs extended (L. Log); left lateral side lying at a half-stomach position (L. Sto); left lateral side lying at a fetal position (L. Fet); right lateral side lying (R. Log); right lateral side lying at a half-stomach position (R. Sto); right lateral side lying at a fetal position (R. Fet.); prone position with head turned left (L. Pr.); and prone position with head turned right (R. Pr.). The no-blanket condition is displayed for illustration and not included in the dataset.

During the experiment, the participants removed all metal-containing clothing or accessories (e.g., belts), shoes, and outerwear. They were instructed to start in the supine position on a bed with a pillow. Then, they were asked to position themselves in nine sleeping postures sequentially, as shown in Figure 2.

(1) Supine (S);
(2) Left lateral side lying with both legs extended (L. Log);
(3) Left lateral side lying at a half-stomach position (L. Sto), bottom leg extended and top leg flexed;
(4) Left lateral side lying at a fetal position (L. Fet), both legs flexed;
(5) Right lateral side lying (R. Log);
(6) Right lateral side lying at a half-stomach position (R. Sto), bottom leg extended and top leg flexed;
(7) Right lateral side lying at a fetal position (R. Fet.), both legs flexed;
(8) Prone position with head turned left (L. Pr.);
(9) Prone position with head turned right (R. Pr.).

When the participants were instructed to perform a specific posture, they could decide to position their limbs and bodies in a manner they found comfortable, as long as it adhered to the defined instructions for the postures. Once the participants confirmed their posture, they were required to remain stationary. The researchers then proceeded to sequentially drape blankets over the participants, ranging from thick to thin. After each blanket was positioned, the researchers paused for five seconds to allow for ambient recording time. After all blanket conditions were tested, a bell signaled the transition to the next posture, and this cycle continued until all nine postures were tested in the three blanket conditions (note: the null blanket condition was not included in our analysis. Figure 2 is just for illustration). The entire process was repeated three times, resulting in three repeated trials. In total, the experiment yielded 5670 data samples (70 participants × 9 postures × 3 blanket conditions × 3 trials) that were manually labeled.

### 2.4. Spatial Radar Echo Map (SREM)

A typical IR-UWB radar data frame is a 2D matrix where each row corresponds to a different radar pulse, capturing the temporal evolution of the scene over time, and each column corresponds to a sample point within a single radar pulse, capturing high-resolution distance information. For each radar sensor, we extracted a data frame at a specific time instance and performed noise cancellation using clutter suppression, achieved through a mean subtraction method as illustrated in Equation (2) [40]:

(2) $$X^{\prime }\left[n,m\right]=X\left[n,m\right]-\frac{1}{N}\sum_{i=0}^{N-1}X[n,i]$$

where *X* is the radar frame with the radar bin *n* and time *m*, and *N* denotes the total number of radar bins.

For each radar frame, the Radar Echo Map Generation algorithm (Algorithm 1) computed the distance from the radar location to every grid point within the predetermined map limits. We then initialized a 2D grid base on the size of the bed. Using the calculated distances, the algorithm identified the nearest radar bins for each grid point and employed interpolation techniques to estimate the radar reflectivity intensity at the specific location.
**Algorithm 1.** Radar Echo Map Generation**Input**: Radar $R_{i}$ defined as arrays of intensity of all radar bins **Output:** Two dimensional intensity Map Q distributed on the bed
*Initialisation:*1:$x_{0}y_{0}=(0,\;0$)2:$x_{N}y_{N}=(90,\;196$)3:maximum detection range=9.87m4:maximum number of radar bias=15365:d=9.87 ∗ 100 / 1536 (Where d is the distance between each radar bin)6:N=int(90/0.643)7:M=int(196/0.643)
*LOOP Process*:8:**for** i in Radar frames $R_{i}$ **do**9: **for** n in $\;x_{0},\;\ldots \;,\;x_{N}$ **do**10:  **for** m in $y_{0},\;\ldots \;,\;y_{M}$ **do**11:$\;\;\;D\left[n\right]\left[m\right]=\sqrt{{\left(X_{n}-x_{r}\right)}^{2}}+\sqrt{{\left(y_{n}-y_{r}\right)}^{2}}$12:$\;\;\;b_{small}=floor(D[n][m]/d)$13:$\;\;\;b_{large}=ceil(D[n][m]/d)$14:$\;\;\;Q[n][m]=Q[n][m]+R_{i}[b_{small}](D[n][m]\;mod\;d)/d+R_{i}[b_{large}](d-D\left[n\right]\left[m\right]mod\;d)/d$15:  **end for**16: **end for**17:**end for**18:return Q[n][m]d: the distance between each radar bin; N: the number of xbins mapped to short edge of bed; M: the number of ybins mapped to long edge of bed; b: radar bin number.

**Figure 3:**
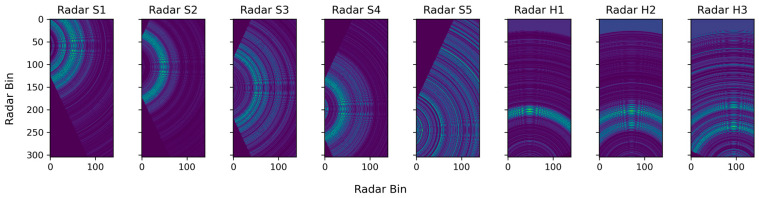
An illustration of Spatial Radar Echo Maps (SREMs) in all radars.

The objective of interpolation is to facilitate the spatial distribution of radar reflectivity values, contingent upon their proximity to the radar source. Upon iterating over all grid points within the defined map boundaries, the radar data can be registered and mapped for a spatial representation. Figure 3 shows the Spatial Radar Echo Maps (SREMs) which were generated from the eight radar sensors, each disclosing a sector-shaped coverage area. This sector shape emerges due to the effective azimuth angle of the radar, spanning from −65 to +65 degrees. It is presumed that the radar signals significantly diminished beyond this azimuth range.

Upon initialization, the algorithm A1 established the map’s boundaries (start and end positions in centimeters), the distance represented by each data bin from the radar, and the number of bins in both the horizontal and vertical directions. It then created stacks of 2D arrays, where each element corresponded to a specific location within the map.

### 2.5. Model Training

**Figure 4:**
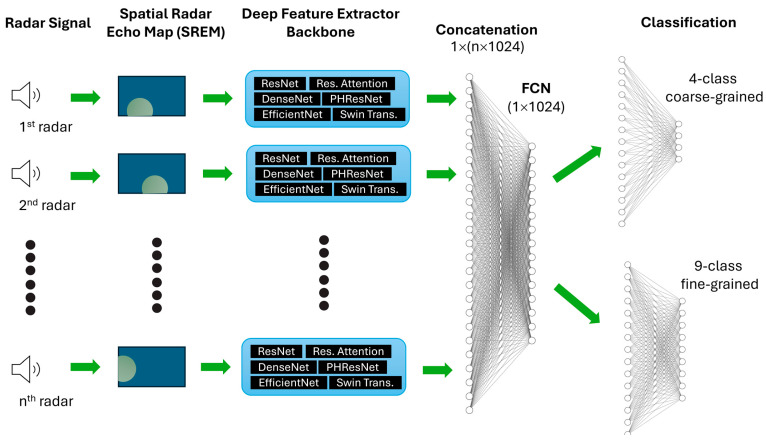
Model architecture of MWCNN with different feature extractors.

We utilized the Multiview Convolutional Neural Network (MVCNN) approach, which was originally used to project a 3D object into multiple 2D images captured from various perspectives [47]. It involved a deep feature extractor on the radar generation maps from each radar, followed by a view pooling operation across all views, and then through fully connected layers for a final classification, as illustrated in Figure 4. In this study, we evaluated the use of ResNet-50 [48], EfficientNet-B0 [49], DenseNet-121 [50], PHResNet-50 [51], residual attention network (Attention-56) [52], and Swin Transformer [53] as the deep feature extractors. PHResNet-50 (Parametrized-Hypercomplex ResNet) is one of the cutting-edge models that facilitates hypercomplex learning for multiview data. The hyperparameters remained at their default values.

The data were split into training and testing set at a 55:15 ratio. Specifically, data from 55 randomly selected participants were used for the model training, while the data of the remaining 15 participants were used for the model testing. Cross-entropy, which acts as the loss function of the model, guides the network to adjust its internal weights to minimize classification errors. We adopted the AdamW, a variant of the Adam optimizer that accounts for the decoupled weight decay regularization. The learning rate was set to 0.001. The betas parameter was a tuple of two values (0.9, 0.999).

### 2.6. Evaluation and Analysis

The performances of the models were evaluated using the accuracy measure, which is defined as the ratio of correct predictions to the number of cases in the testing set. In addition to the full analysis (i.e., 9-class classification), we also evaluated 4-class coarse-grained classification to provide more insights on the performances of the models. This involved categorizing the nine original classes into four coarse categories: supine, left, right, and prone. The four-class classification model was then trained and evaluated independently. The categorization of the nine classes was as follows:

(1) Supine: S;
(2) Left: L. Log, L. Sto, L. Fet;
(3) Right: R. Log, R. Sto, R. Fet;
(4) Prone: L. Pr, R. Pr.

Once the optimal model was identified, we retrained and retested it using data from various numbers and placements of radar sensors. However, it is important to note that we did not explore all possible combinations of radar sensor numbers and placements. Instead, we pre-planned several combinations and quantities based on specific premises, which are detailed in Section 3. In total, we experimented with 22 different settings involving various numbers and combinations of radar sensors. We decided to use 4-class classification scheme for the radar configurations’ evaluation, since this approach offers a more interpretable means to understand which radar configurations contributed more significantly to the model performance.

## 3. Results

### 3.1. Performance of Deep Learning Models

As shown in Table 2, DenseNet-121 consistently outperformed the others with accuracies of 0.534, 0.714, and 0.804, for the nine-class and four-class classifications, respectively. EfficientNet-B0 also demonstrated a competitive performance, achieving an accuracy of 0.775 for the four-class classification. Attention-56 managed to achieve an accuracy of 0.469 in the coarse-grained classification, but it failed to converge in the fine-grained classification. Unfortunately, the Swin Transformer model did not converge in any of the classification tasks.

### 3.2. Performance of Different Radar Arrangements and Placements

DenseNet-121, ResNet-50, EfficientNet-B0, and PHResNet-50 were selected for further analysis of the radar arrangements and placements. Table 3 shows the impacts of varying radar configurations on the accuracy of the four-class posture classification. The baseline configuration (#1) on all eight radars achieved an accuracy of 0.804. This performance did not weaken much when removing one and two radars (#2 to #7), showing accuracies of 0.794 and 0.771 for DenseNet-121, respectively. When only six radars were retained (#5 to #7), the placement of the radars played an important role. Interestingly, the performance of a specific configuration (#5) surpassed the baseline with an accuracy of 0.809 for DenseNet-121, while that of #7 was very near to the baseline with an accuracy of 0.803. This finding showed that the H_L_ radar played an important role in the model performance. The variations in this performance became greater when the number of radars were further reduced.

Table 4 compares the average accuracies across the various models with different numbers of radars. When only six radars were used, DenseNet-121 revealed an average performance of 0.799, which is comparable to the baseline configuration with eight radars. Although ResNet-50 generally outperformed DenseNet-121 across the other radar configurations, DenseNet-121 consistently showed an optimal performance, indicating its suitability for adoption.

## 4. Discussion

The objective of this study was to determine the ideal quantity and positioning of radar sensors for sleep posture estimation, thereby laying the groundwork for the optimal sensor configuration in this application. We incorporated eight radars into the baseline setup, with three positioned at the headboard and five along the side. In order to accommodate the multiple radar sensors, we introduced an innovative data fusion method for generating radar maps, the Spatial Radar Echo Map (SREM), and ingeniously utilized the Multi-View Convolutional Neural Network (MVCNN).

Multimodal data fusion has attracted significant attention in recent studies. The integration of diverse data sources may enhance the predictive accuracy and robustness in various applications, specifically for situations where time series data are the major sensory data type [54,55]. We employed a data fusion approach in this study, since our study utilized multiple IR-UWB radars as the primary devices for sleep posture recognition.

Moreover, we employed sensor removal to isolate the influences of individual radars within the chosen model. We opted to focus on the best-performing model (DenseNet-121), since this enables a more precise attribution of performance variations to the removed sensors.

In regard to sensor placement, for the head radars, positioning a single one on the left was both crucial and adequate. A lack of all head radars led to a significant decrease in prediction accuracy. However, adding more radars could potentially diminish this accuracy slightly. This could be attributed to the possibility that extra radars at the shoulder may not provide informative data, but rather contribute to noise. Nevertheless, we decided to maintain the central radar because of its better exposure and alignment with our existing study.

Increasing the number of side radars generally improved the prediction accuracy. This could be attributed to the fact that all radars were essential for identifying the fine-grained features in postures, such as limb placement, which helps to distinguish between postures like the log, fetal, and half-stomach positions. If we aim to limit the number of side radars to four or three, the optimal configuration involves removing the central radars for the head edge and retaining those focused on the upper-body regions for the side edge. It appears that radars targeting the upper-body region are more effective in estimating sleep postures in general. We initially hypothesized that the baseline configuration would yield the highest accuracy. However, configurations (#6) and (#8) demonstrated comparable accuracies, despite the removal of two head radars. This finding suggests that there could be the presence of a ceiling effect when an adequate number of side radars are employed. In our setup, all side radars were placed equidistantly to ensure uniform exposure, which may have contributed to this ceiling effect. Future research should explore not only the number of radars used, but also the interval of their placement.

We compared the performance of our system to that of existing studies (Table 5). Zhou et al. [56] utilized an FMCW radar system with a CNN incorporating an Inception-Residual module across eight sleep postures, with overall accuracy of 87.2%. Piriyajitakonkij et al. [40] employed the Xethru X4M03 radar and SleepPoseNet, achieving an accuracy of 73.7 ± 0.8% across four sleep postures. Islam and Lubecke [57] used a dual-frequency monostatic CW radar with multiple classifiers (KNN, SVM, and Decision Tree) and reported an accuracy of 98.4% for this dual frequency. Adhikari [58] used a Texas Instrument IWR1443 radar with the Rest Network, a customized CNN, achieving an 80.8% accuracy across five postures without blankets. Our previous study [44] utilized the spatial–temporal features of continuous radar frames, employing various models, including the Swin Transformer with the Xethru X4M03 radar, and achieving up to an 80.8% accuracy for four sleep postures with blankets. In this study, we decided to utilize the spatial features of single radar frame, which could enable real-time application. Using the Xethru X4M03 radar and DenseNet121 model, we classified four sleep postures with three blanket conditions, achieving the highest accuracy of 80.9%. This indicates that our approach is comparable to or slightly better than previous results, despite the additional complexity of three blankets.

There were some limitations in this study. While our proposed data fusion technique using a radar generation map reinforced the presentation of spatial information, temporal information might also be useful in estimating sleep postures by their reasonable transitions. The quasi-periodic oscillations in radar signals contributed by vital signs might facilitate attention to the torso region and improve the performance of posture estimation [44]. The constraint of data size was another limitation. Deep learning models generally require substantial amounts of data to achieve the optimal performance and model convergence, especially those using complicated models.

In our study, we observed that the Swin Transformer did not converge in both the fine-grained and coarse-grained classifications, while Attention-56 did not converge in the fine-grained classification and underperformed in the coarse-grained classification. Both models belong to the class of attention-based models, which are fundamentally different from convolutional networks. Convolutional networks primarily focus on local surrounding spatial features through filters. However, implementing the attention mechanism also comes with a trade-off: a significant increase in the number of parameters (Table 2). This, in turn, necessitates training with more data, especially data rich in latent information, for the attention module to effectively capture these subtle relationships. However, if the dataset lacks sufficient non-local features or contains repetitive long-range features, the Transformer model may fail to converge. In our study, the signature of the feature in the radar spatial map was localized, indicating a lack of non-local relationships. This could be a potential reason for the non-convergence of the models. Furthermore, our radar map represents a single instant without any time features. This means we could not track the movements of individuals over time to facilitate the attention mechanism. If there was a time domain, it might introduce some non-local time features that could potentially aid the convergence of Transformer class models.

Prior research on sleep posture recognition has often prioritized the expansion of recognized postures, in addition to the presence of blankets. However, the orientation and covering style of these blankets are important, yet frequently neglected, factors that might influence accurate classification. Our study prioritizes real-world applicability by acknowledging the variability in self-covering behaviors during sleep. To address this, we will incorporate scenarios with diverse blanket orientations and covering methods as part of our external testing procedures. We posit that this inclusion will enhance the generalizability of our proposed sleep posture recognition model.

## 5. Conclusions

This study identified the optimal combination of radar quantity and placement, starting with eight radar sensors, three at the headboard and five along the side. The left head radar was found to be essential for achieving accurate posture estimation, while the performance generally improved with an increase in the number of side radars. A cost-effective compromise could be achieved by either omitting the central side radar or retaining the three radars focused on the upper body. A novel data fusion strategy, termed Spatial Radar Echo Map (SREM), was introduced in conjunction with the Multi-View Convolutional Neural Network (MVCNN). Using DenseNet-121 in MVCNN and retaining one central head radar, the accuracy of the four-class coarse-grained posture estimation was 0.809 when we retained all side radars, 0.779 when we removed the central side radar, and 0.753 when we retained the three side radars at the upper body. Future research directions can consider different orientations and blanket covering styles to enhance the model’s generalizability.

## Figures and Tables

**Table 1:** Parameters of the IR-UWB radar sensors [46].

Parameters	Values
Transmitter Frequency (Tx)	7.29 GHz
Transmitter Bandwidth	1.4 GHz
Pulse Repetition Frequency	15.188 MHz
Sampling Frequency	23.328 GHz
Range of Elevation angle	−65° to +65°
Range of Azimuth angle	−65° to +65°
Bin Length	0.00643 m
Detection Range	0.0–2.0 m
Bin Resolution	312 bins per radar frame
Frame Rate	20 frames per second
Transmission Power	6.3 dbm

**Table 2:** Accuracy of different deep learning models as deep feature extractor in 9-class and 4-class sleep posture classification, and the number of parameters of each model.

Model	Nine-Class Fine-Grained	Four-Class Coarse-Grained	Number of Parameters
ResNet-50	0.496	0.721	2.05B
EfficientNet-B0	0.454	0.775	42.55M
DenseNet-121	0.534	0.804	64.02M
PHResNet-50	0.468	0.723	2.08B
Attention-56	NC	0.469	4.62B
Swin Transformer	NC	NC	11.06B

NC: model did not converge.

**Table 3:** Four-class coarse-grained classification accuracy of different radar configurations using DenseNet121, ResNet-50, EfficientNet-B0, and PHResNet-50.

Conf	*N*	*S*_1_	*S*_2_	*S*_3_	*S*_4_	*S*_5_	*H_L_*	*H_C_*	*H_R_*	DenseNet121	ResNet-50	EfficientNet-B0	PHResNet-50	Implications
#1	8	✕	✕	✕	✕	✕	✕	✕	✕	0.804	0.721	0.775	0.723	Baseline
#2	7	✕	✕	✕	✕	✕	✕	✕		0.703	0.774	0.688	0.738	Head radar removal
#3	7	✕	✕	✕	✕	✕	✕		✕	0.794	0.787	0.758	0.729	
#4	7	✕	✕	✕	✕	✕		✕	✕	0.771	0.771	0.662	0.728	
#5	6	✕	✕	✕	✕	✕	✕			0.809	0.781	0.702	0.750	Retain single head radar
#6	6	✕	✕	✕	✕	✕		✕		0.785	0.760	0.684	0.735	
#7	6	✕	✕	✕	✕	✕			✕	0.803	0.760	0.641	0.722	
#8	5	✕	✕	✕	✕			✕		0.675	0.758	0.652	0.698	Side radar removal with central head radar retained
#9	5	✕	✕	✕		✕		✕		0.674	0.755	0.662	0.707	
#10	5	✕	✕		✕	✕		✕		0.779	0.720	0.735	0.698	
#11	5	✕		✕	✕	✕		✕		0.750	0.728	0.715	0.708	
#12	5		✕	✕	✕	✕		✕		0.770	0.744	0.725	0.691	
#13	5	✕	✕	✕	✕	✕				0.661	0.708	0.736	0.707	No head radar
#14	4		✕		✕		✕		✕	0.592	0.721	0.698	0.662	2 head and side radars
#15	4	✕		✕		✕		✕		0.612	0.712	0.709	0.687	3 side radars with central head radar retained
#16	4			✕	✕	✕		✕		0.728	0.676	0.684	0.653	
#17	4		✕	✕	✕			✕		0.735	0.724	0.696	0.669	
#18	4	✕	✕	✕				✕		0.753	0.722	0.725	0.690	
#19	4	✕			✕	✕		✕		0.702	0.668	0.678	0.625	
#20	4	✕	✕			✕		✕		0.748	0.707	0.709	0.691	
#21	3		✕		✕			✕		0.550	0.672	0.678	0.656	Settings of our previous study [44,45]
#22	2			✕				✕		0.594	0.649	0.633	0.613	

N: number of radar sensors. S_1–5_ denote radar sensors arranged from cranial (S_1_) to caudal direction. H_L,C,R_ denote radar positions at the left, center, and right of the headboard. #1–#22 denote configuration number 1–number 22. ✕ indicates the radar to retain.

**Table 4:** Average accuracy of different number of radars used across DenseNet-121, ResNet-50, EfficientNet-B0, and PHResNet-50.

N	DenseNet-121	ResNet-50	EfficientNet-B0	PHResNet-50
8	0.804	0.721	0.775	0.723
7	0.756	0.777	0.703	0.732
6	0.799	0.767	0.676	0.736
5	0.718	0.736	0.704	0.702
4	0.696	0.704	0.700	0.668
3	0.550	0.672	0.678	0.656
2	0.594	0.649	0.633	0.613

N: number of radar sensors.

**Table 5:** Comparison of accuracy performance with existing studies.

Author (Year)	N_p_	N_s_	N_b_	Radar Hardware	Best Model	Accuracy
Zhou, et al. [56]	3	8	0	FMCW radar system	CNN w/Inception-Residual module	87.2%
Piriyajitakonkij, et al. [40]	38	4	0	Xethru X4M03	SleepPoseNet: a Deep CNN w/MW Learning	73.7 ± 0.8%
Islam and Lubecke [57]	20	3	0	Dual-frequency monostatic CW radar	Decision Tree	Dual: 98.4%
Adhikari and Sur [58]	8	5	0	Texas Instrument IWR1443	Rest Network, a customized Deep Convolutional Neural Network	95.6%
Lai, et al. [44]	30	4	1	Xethru X4M03	Swin Transformer	80.8%
This study	70	4	3	Xethru X4M03	MWCNN w/DenseNet121	80.9%

CNN: convolutional neural network; MW: Multiview; N_b_: Number of blanket conditions; N_p_: Number of participants; N_s_: Number of sleep postures to be classified; w/: with.

## Data Availability

The program, model codes, and updates presented in this study are openly available in GitHub at https://github.com/BME-AI-Lab?tab=repositories (accessed on 31 July 2024). The video/image dataset are not available due to subject confidentiality issue.
